# Health and social care experience and research perception of different ethnic minority populations in the East Midlands, United Kingdom (REPRESENT study)

**DOI:** 10.1111/hex.13944

**Published:** 2023-12-21

**Authors:** Winifred Ekezie, Shabana Cassambai, Barbara Czyznikowska, Ffion Curtis, Lauren L. O'Mahoney, Andrew Willis, Yogini Chudasama, Kamlesh Khunti, Azhar Farooqi

**Affiliations:** ^1^ National Institute for Health and Social Care Research (NIHR), Applied Research Collaboration East Midlands (ARC EM) Leicester UK; ^2^ Diabetes Research Centre University of Leicester Leicester UK; ^3^ Centre for Ethnic Health Research University of Leicester Leicester UK; ^4^ Department of Sociology and Policy Aston University Birmingham UK; ^5^ Liverpool Reviews and Implementation Group (LRiG) University of Liverpool, Institute of Population Health Liverpool UK; ^6^ HRB Clinical Research Facility & School of Public Health University College Cork Ireland; ^7^ Leicester Real World Evidence University of Leicester Leicester UK; ^8^ Leicester City Clinical Commissioning Group (CCG) UK

**Keywords:** ethnic minorities, health inequalities, health research, research priorities, social care, United Kingdom

## Abstract

**Introduction:**

Ethnic minority populations experience significant health and social care disparities; despite experiencing a greater burden of diseases, these groups are underrepresented in health and social care research. Consequently, related research can be less applicable to these population groups. The REPRESENT study aims to explore the health and social care experiences of ethnic minorities and other minoritised populations, their research interests and appropriate research practices.

**Methods:**

Focus groups and semistructured interviews were conducted between May and September 2022 with members of a number of ethnic minority communities in England. Data were audio recorded, transcribed and thematically coded using NVivo 12. Rigour was determined through extensive sampling, iterative data collection and analysis.

**Findings:**

Fifty‐two ethnic minority members were engaged in group interviews and one‐to‐one interviews. Participants included representatives of the following groups: African Caribbean, Eastern European, Gypsy Travellers, Lesbian, Gay, Bisexual, Transgender, Queer, Intersex and Asexual+, Refugee/Asylum Seekers, Somali and South Asian communities. Interviews were also conducted with ethnic minority healthcare providers and researchers. Three overarching categories were identified: health information, medical service experiences, health and social care concerns and health research. Health and social care services challenges were mostly attributed to discrimination, delayed services, poor cultural relevance and language and cultural barriers. The most influential information sources were local community organisations and word‐of‐mouth. The main health and social care concerns were chronic long‐term health conditions, mental health, maternal health and child development. Recommendations for research involved understanding the motivations for participation, improving communication and empowering communities. Top research priorities were long‐term health conditions, health promotion and education, early care interventions and understanding community needs.

**Interpretation:**

Discrimination and bias in health and social care provision have severe implications for worsening ethnic health inequalities. Healthcare commissioning authorities and policymakers can leverage the preference of ethnic minority groups for pharmacy services and community organisations to improve access to care. Improving research interest and engagement requires understanding individual community needs, community sensitivity, research relevance and cultural appropriateness.

**Patient or Public Contribution:**

Members of ethnic minority Patient and Public Involvement and Engagement group and Community Advisory Board supported the REPRESENT study design, conceptualisation and report development.

## 1 INTRODUCTION

Ethnic minority populations are often underrepresented in health and social care research, despite experiencing a more significant burden of diseases.^1^ These disparities in health research were particularly highlighted in the recent COVID‐19 pandemic, which disproportionately affected vulnerable and marginalised populations, including ethnic minorities in the United Kingdom; however, representation of these groups in clinical trials was significantly low.^2^ Similarly, poor ethnic minority representation has been observed in several other health research areas, including cancer, diabetes and cardiovascular disease.^3^, ^4^, ^5^, ^6^ The consequence of this is that medical and social care research, which informs guideline recommendations and everyday clinical practice, is less generalisable and applicable to ethnic minority populations. More diverse research cohorts are essential for promoting equitable research and would ensure reductions in health outcome disparities.^7^ Hence, considering the health and social care concerns and research priorities of ethnic minorities could encourage their interest and participation in research, which is needed for investigating health interventions.^8^, ^9^, ^10^

The United Kingdom (UK) presents a diverse ethnic minority population,^11^ research involving minority groups can increase understanding of the aetiology and management of long‐term health conditions.^6^ This has overall long‐term benefits to better quality of life, through enhanced health management and also serves as an economic factor to reduce the acute burden on national health services through mitigating worsening of health conditions through better understanding and management.^12^ Therefore, increasing diversity awareness has implications for policy and practice, improved access and dialogue with specific communities, and identification of culturally appropriate approaches to managing illness. Reasons for the exclusion of minority ethnic groups are complex due to several factors, with significant barriers to participation among minority groups in the research process, including a poor understanding of their interests, needs, preconceived reservations and resources to engage them effectively^13^; thus, priority setting is essential. Identifying relevant research priorities starts with understanding the experiences and needs of communities, and awareness of these will assist researchers and policymakers in addressing areas of greatest need, and best practice entails identifying and prioritising research important to stakeholders.^14^ Current evidence shows most research priority settings do not report ethnic minority involvement in research agenda development and often lack research process transparency.^13^, ^15^ Ensuring equity‐focused research priority setting requires the involvement of patients, caregivers and the public, including ethnic and marginalised minority groups.^14^, ^16^

Strategies to enhance ethnic and other minoritised population recruitment are essential, however practical guidance is scarce. Research is unlikely to deliver useful findings without good practices such as inclusivity of stakeholders in research prioritisation, implementation and evaluation.^15^ This means that identifying opportunities to improve access for communities is crucial. Developing an understanding of the research interests and preferred engagement procedures of ethnic minorities would ensure future research reduces the under‐representation of ethnic minorities and increases the generalisability of research findings. This current study aimed to explore the health and social care experiences of ethnic and other minority populations in the United Kingdom and identify priority areas for future research.

## 2 METHODS

### 2.1 Study design

The REPRESENT study is a programme of work established to investigate the health and social care research priorities among ethnic minority populations in the United Kingdom.^17^ The current study presents feedback from qualitative data collected from ethnic minority communities in the East Midlands between May and September 2022.

### 2.2 Study settings and participants

Ethnicity can be defined as a ‘social group a person belongs to, and either identifies with or is identified with by others, due to a mix of cultural and other factors including language, diet, religion, ancestry and physical features traditionally associated with race’.^18^ This use of ethnicity allowed us to include inequalities experienced within broader racial groups—for example, White Gypsy Travellers (GT) communities. We also included other minoritised populations within the ethnic minority community (e.g., Lesbian, Gay, Bisexual, Transgender, Queer, Intersex and Asexual [LGBTQIA]+ representatives).

All ethnic groups except the White British group were included in this study. Recruitment of participants was done through existing Patient and Public Involvement and Engagement (PPIE) networks, social media and snowballing. Purposive sampling and snowballing were also used to identify participants, including using local ethnic minority networks. To reach a breadth of participants, the Centre for Ethnic Health Research (CEHR) team utilised their existing networks to engage diverse ethnic minority groups with varying accessibility needs. This included using face‐to‐face, online and remote platforms for recruitment, which was facilitated through community networks and forums, including voluntary sector and faith organisations. Furthermore, the study was advertised through the CEHR social media channels and WhatsApp groups.

Participants included ethnic minority community groups, healthcare providers (HCPs) and researchers. Participants were recruited from across the East Midlands region in the United Kingdom (Lincolnshire, Northamptonshire, Derbyshire, Nottinghamshire, Leicestershire and Rutland). The sampling strategy was designed to include all protected characteristics within the Equality Act of 2010.^19^

The group and individual interviews were conducted by two authors (B. C. and S. C.) with extensive experience working with minority communities. The focus group structure considered cultural and linguistic factors (e.g., single‐sex groups and language support) and availability. Data were collected virtually using the Zoom platform and face‐to‐face engagement based on participant availability and preference. Eligible participants were contacted at least 2 weeks in advance, and consent was obtained from each participant before data collection. Ethical approval for the study was received from the University of Leicester Research Ethics Committee.

### 2.3 Data collection and analysis

The interview topic guide covered topics on health care experience, health and social care concerns, and health research (see File S1). The topic guide was co‐produced and pretested with community members; in addition, specific questions were added for researchers and HCPs.

All data collection was conducted in English; however, ad hoc phrases were translated during the sessions with Polish and Somali communities where required. On average, group interviews lasted 95 min, and interviews 70 min. Audio recordings were transcribed verbatim directly to English by two researchers (B. C. and S. C.), and W. E. verified each transcript for quality, consistency and accuracy. A thematic approach based on the topic guide was used to analyse the data using Nvivo 12.0 software. The analysis followed the six‐stage process proposed by Braun and Clarke: familiarisation with the data, generating initial codes, searching for themes, reviewing initial themes, defining and naming themes and producing the report.^20^ The transcripts were coded by W. E.; these were developed and discussed with the wider team to enhance identified transparency, consistency and promote reflexivity.^21^ Some research team members (B. C. and S. C.) have previously been involved in face‐to‐face contact with a few study participants during previous projects, which may have influenced their interactions with participants. However, the initial data analysis was carried out by another member not directly involved with the participants, and all team members reviewed the findings, so this minimised any presumptions in the data interpretation.

### 2.4 Patient or public contribution

Members of ethnic minority PPIE groups and the CEHR supported the REPRESENT study design, conceptualisation and recruitment. Seven community advisory board members representing the different Equality Act categories reviewed the topic guide to ensure ease of understanding, cultural relevance and sensitivity. Furthermore, four study steering committee community members previewed the findings to ensure cultural accuracy.

## 3 RESULTS

A total of 52 people participated in the focus groups and one‐to‐one interviews. Nine focus groups were conducted with 45 participants. Individual interviews were held with seven people representing unpaid carers, nurses/midwives, general practitioners (GPs) and researchers (see Table 1).

**Table 1:** Summary of study participants.

Data collection approach	Cohort	Age range	Gender	Number of participants
Focus group (*n* = 45)	African Caribbean	45–70	Females—4 Male—1	5
	Eastern European	35–50	Females—2 Males—1	3
	Gypsy Travellers	27–40	Females— 3	3
	Lesbian, Gay, Bisexual, Transgender, Queer/questioning, Intersex, Queer/questioning, Asexual and others (LGBTQIA+)	28–58	LGBTQ	4
	Refugee/Asylum Seekers	25–45	Females—3 Males—4	7
	Somali	25–55	Females—6 Males—2	8
	South Asian (mixed gender)	32–60	Females—3 Males—3	6
	South Asian men	37–55	Males	4
	South Asian women	25–45	Females	5
Interviews (*n* = 7)	Carer (family member)	70	Female	1
	Researchers (paramedic/occupational therapist)	Not disclosed	Male—1 Female—1	2
	Nurse/midwife	45	Female	2
	GP/retired GP	55–75	Male—1 Female—1	2

Abbreviation: GP, general practitioner.

**Figure 1:**
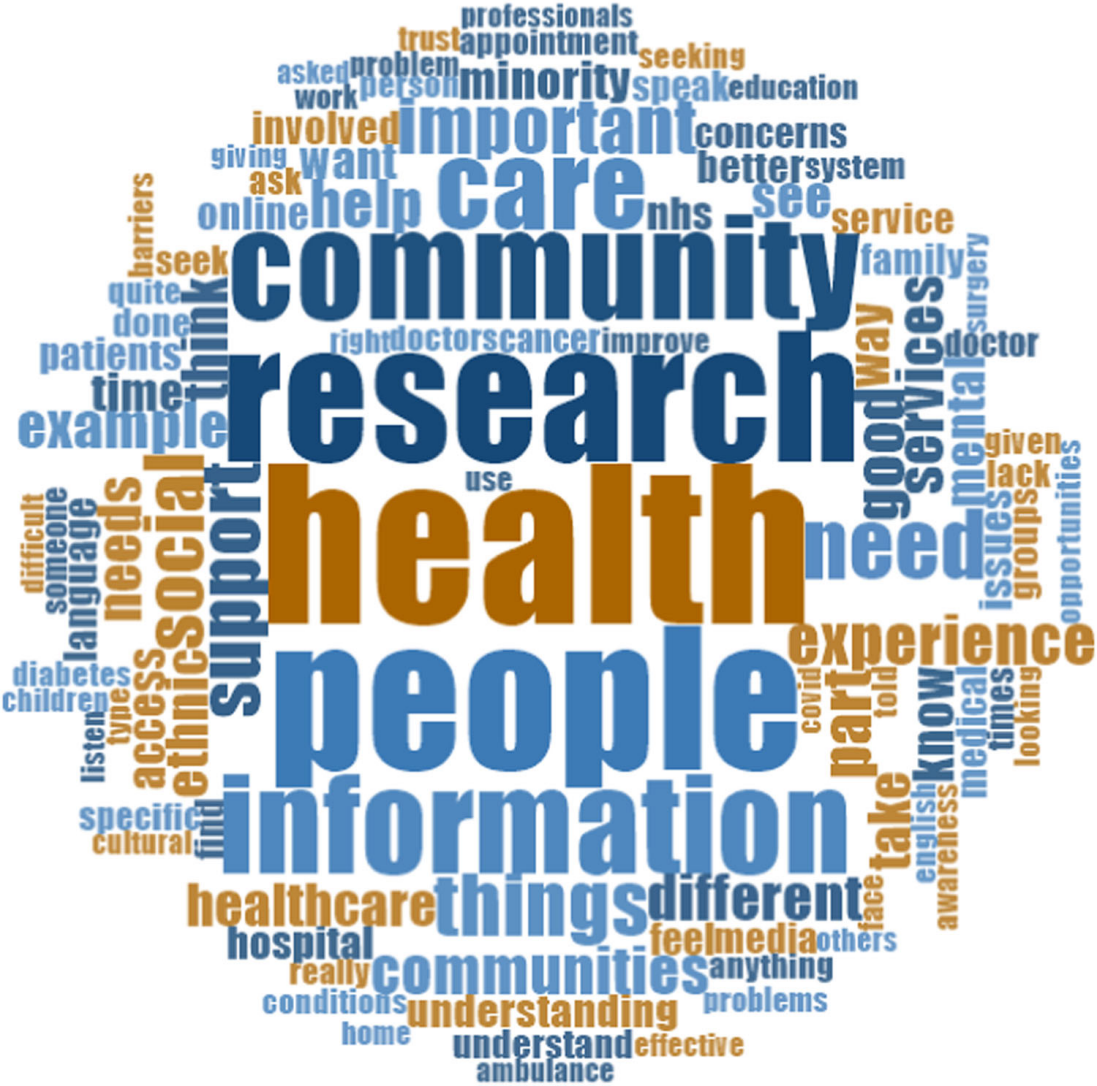
Word cloud of 100 most commonly used words by study participants.

Figure 1 presents a word cloud, created using Nvivo 12 software, of the most common 100 words participants used. These words illustrated participants' reflections on the importance of social and health care in the community through health research for people, information and experiences. The word cloud also highlights priority health conditions of interest, such as cancer, diabetes and COVID‐19, as well as critical issues for consideration, for example, access, language and cultural factors. The experiences across all the participant ethnic groups were explored; three overarching themes were developed from the thematic analysis of the data, as summarised in Table 2. The first section addresses the first study aim, and the last theme addresses the second aim.

**Table 2:** Summary of identified themes and subthemes.

Theme	Subtheme
Health information and healthcare seeking actions	
Medical, health and social care service experiences	Access to services
	Discrimination
Health research	Research understanding, barriers and motivators
	Health service improvement recommendations
	Research recommendations

### 3.1 Health information and healthcare seeking actions (Aim 1)

The most reported source of information was via online platforms, especially the Google search engine. At a community level, common information sources were word‐of‐mouth, local community organisations and networks, peer‐to‐peer support, family, friends and local pharmacies and GPs. Although online sources were common, several participants stated it was often confusing, and the information was sometimes exaggerated and unreliable, causing increased anxiety. Participants also stated that information about certain conditions could not be found online, for instance, guidance on managing a disabled child.

…having a special needs child, there are few things you cannot find on the internet related to health, a disabled person… There is a lot of information online, but for some specific information, you need a specialist advice, an expert… (FG, South Asian men)

Thus, community organisations were considered good for finding information as they provided platforms for members to meet and ask questions directly from people experiencing similar issues. To reinforce this, local organisations used different strategies to initiate interactions between health providers and community members, as explained by a participant:

We did a community initiative for ethnic minority groups based at Leicester General Hospital, a meeting with a GP—we gathered issues from the community and discussed with local GP … looked at what can be done and shared the feedback back with the community. (FG, African Caribbean)

Reasons for seeking information sources outside official health channels were related to difficulty in getting appointments to see a GP, insufficient time to discuss more than one health issue and lack of cultural understanding. They also highlighted receiving poor health assessment and information from their GPs, with little to no information on some issues, and sometimes contradictory information compared to what was obtained online, as described by a participant below:

…I was told by a staff member there that I am prone to sickle cell because of my heritage… I Googled, I did find that yes people from certain heritage are more likely to be diagnosed with sickle cell. However, I am not really from South Asian background, I am from a different part of Asia (Middle Eastern) … in the research online, the prevalence in my ethnic group is not as high as I was told, but the person who told me, made an assumption about me‐ quite poor assessment and information given. (FG, LGBTQIA+)

Despite online information being common, a few groups were still excluded, e.g. those without internet access. A Polish participant explained how this communication gap and language challenges contributed to the delayed presentation of health issues at healthcare facilities.

…many Poles, mainly those with limited language skills, in low‐income and low‐skills level jobs … tend to avoid contact with healthcare services until they are ill … when things are bad … due to fear of losing jobs … they fear potentially being asked to attend other appointments, they keep asking you to do other things, sometimes you regret why you accessed in the first place. (FG, Eastern European)

Improving community health communication was considered a top priority for adequate health‐seeking action. The key recommendation was to consider the right person to present the information carefully. This included finding someone from that same ethnic community known by the community, having bilingual HCPs and community champions/ambassadors, and more direct interactions between the health services and communities, such as speaking on community radio and face‐to‐face conversations at community centres. Other crucial factors highlighted were privacy and convenient access to information. Privacy, providing information in a more private context, with one‐to‐one support, individually, was especially highlighted in relation to mental health and addiction‐related issues, as shown:

When it comes to seek information/support on some specific issues (mental health, substance misuse), people will seek information in more private context, one‐to‐one support, individually. (FG, Eastern European)

### 3.2 Medical, health and social care service experiences

A range of health issues affected ethnic minority populations, but no distinctive issues were unique to specific community groups. The most common chronic conditions were cancer, hypertension, diabetes, dementia, autism, musculoskeletal pain and dental issues. All groups reported mental health challenges, and participants stated this was rising rapidly within their communities, yet there was limited awareness and support for prevention and management. Two factors underpinning these issues were access to services and discrimination.

#### 3.2.1 Access to services

Factors included in this category were time, appointment availability, language, and how they affect different groups, for instance, parents with caring responsibility and the older generation. The most reported challenge was language, especially among the older population. In addition, some participants pointed out that men were less inclined to speak out about their challenges.

Medical professionals use a lot of jargon and it's off‐putting. If English is not the first language, then it's really difficult. Informed decisions couldn't be made due to language barriers as procedures are in alternative language… Some HCPs may feel that it may be offensive to share leaflet in another language… Men generally won't ask questions and go with the doctor's recommendations. (Nurse)

Quite a few participants reported challenges with getting appointments, contributing to the delayed presentation of cases and complications. This was also compounded by a generally short consultation time with GPs. Several participants expressed frustration with the emergency service waiting times, stating they were incredibly long, up to 10 h. After seeing HCPs, some participants still reported dissatisfaction with the care received, especially when told no actions were required. Overall, participants felt that priority needs were often not addressed. An example is a Refugee/Asylum Seeker who reported having health issues developed in their home country but did not receive needed care when complaints were made early until it became an emergency.

I went to my GP many times, complaining about pain… He did not give me anything, apart from telling me to drink ginger tea… My pain started in Sudan, and I was telling them that. Only painkillers given—no checks. After being admitted to A&E, they finally did something … a scan and said I will need an operation. Why they do not help when you go early?… You have to go really badly before support is given. (FG, Refugee & Asylum Seekers)

As a consequence of the complexity of access to care, participants stated conditions such as chronic pain were often neglected, and major chronic conditions like cancer and diabetes often became more complicated due to lack of screening, delayed services, inadequate management and lack of awareness. Hence, early intervention was pointed out as an important area of consideration in ethnic minority communities and a fundamental first step to managing health and social care conditions. Nevertheless, these were influenced by various factors, primarily culture, fear, distrust, misconceptions and geographic location. Some communities reported difficulty knowing where to access needed information and support, but language barriers were a major associated factor. The impact of language on early intervention is described by a participant who lost a child due to poor communication:

Language barrier in health can be a big problem … we did not fully understand things about our child, we lost our child, the awareness was not there. We did not have the language skills to be able to understand the full story. For ethnic minorities, having language support is really important. (FG, South Asian men)

#### 3.2.2 Discrimination

Some participants indicated that they were often ignored, dismissed, judged, received inadequate medical support and were treated rudely and differently from others, especially those from White ethnicities, due to their language and skin colour. A Somali participant shared an example of this:

It depends on the person you are seeing. It can be a rude person who does not like your skin colour. Yes, they are professionals, but the way they act it would show that they do not like you … one GP can be good, and another not. Some will only see you for 2 min. Others will give you time, respect. (FG, Somali)

In addition to the generally perceived discrimination, unpaid family carers described being ignored. This included perceived stereotyping by HCPs who often did not acknowledge the opinions of carers, as described by a participant who acted as a carer for their spouse.

…even though I was the wife of the patient. Hospitals not listening to the wife, having to ask the patient, even though patient is clearly not coherent and is unwell. It's very dated and insulting to a carer. (Carer)

Besides ethnicity and service issues, sexual discrimination and other biases were also reported. An LGBTQIA+ community member described how they preferred health services provided by pharmacists compared to nurses who often stereotyped and viewed them with ‘judgemental attitudes’.

Pharmacies are patient‐focused … sensitive to your needs, responsive, and prompt … do not judge you. Contrary to nurses and their judgemental attitudes … you try to speak to them about certain things (e.g., sexuality), they give you these looks … judge you, stereotype you; assuming that you are of certain sexuality. When you correct them, you see the looks on their faces—the way they judge you. (FG, LGBTQIA+)

The lack of mental health awareness, in addition to poor understanding of symptoms, was influenced by racial/cultural discrimination (e.g., general labelling of vocally expressive and loud African Caribbean people as ‘mental health people’) and gender variance. Among women, post‐natal depression was high among new mothers, while males were least likely to talk about mental health. A South Asian participant explained this was because mental health was considered a ‘taboo’, and men were ashamed to express their emotions. A GT specified males between 16 and 40 years were of most concern as it contributed to higher incidences of alcohol and drug use and suicide.

Mental health and men is a big thing. Depression and anxiety. Community pressures, it gets to them. They do not want to talk about this to their wife or men around them because they do not want to be a laughing stock. Therefore, they bottle up. Before you know it, it is too late. Lack of support/understanding leads to three things: alcohol, drugs and suicide… 16‐40 years old men have the biggest problem. Men around 50s are not so impacted because their life is more settled/established. (FG, GT)

General interaction with social services was limited due to discrimination and poor previous experiences with health visitors—especially among GTs who have a unique nomadic and close community lifestyle, which sometimes concerned social service providers. It was also pointed out that social care staff were not well trained and lacked sufficient skills to manage certain conditions properly, resulting in some participants taking on most of the caring burden. A father of a child with a disability shared an experience of this.

I have a disabled daughter… the staff from caring agencies do not have the skills required… They do not look at the patient's needs … and each person is different… Agency staff [care agencies] need to be more trained, improve their services. (FG, South Asian Men)

### 3.3 Health research (Aim 2)

#### 3.3.1 Research understanding, barriers and motivators

Table 3 summarises the participants' understanding of research, the barriers and motivators for participating in research, and suggestions on areas to encourage more active participation.

**Table 3:** Summary of research understanding, barriers and motivators subthemes.

Subthemes	Feedback/suggestions	Supporting quotes
Understanding of research	Good perceptions of research due to previous participation	I understand health research as improving things, making things better for people and the whole society, communities, helping others… (South Asian men) …Like the research about Black women and mortality rate. We know in the Black community, there are more deaths because of the way babies are delivered, home births… (Somali)
	Knowledge of different types of research	Talking research: focus groups, and discussion. But also taking part in research—sometimes you get letters through the post telling you/inviting you to take part in research/to be part of the sample/research group. (South Asian women) Well, there's qualitative and quantitative. You have randomised control trials, systematic reviews. These are all gold standards… (Midwife)
Barriers to research participation	Trust in who is conducting the research	For people who are born in Caribbean means ‘you are going to be used/they are going to use me’, a guinea pig. You do not get anything back when it finishes. Therefore, I am reluctant to be involved. One side benefits (African Caribbean) I will not trust private companies and research. I will trust more Universities, public institutions‐based research. (Eastern European)
	Participants struggle to open up in groups	Participants were asked to be open or share their feedback—I felt people were not open, not comfortable to speak up; honesty of answers—without offending people. (LGBTQIA+)
	Lack of education on research participation	Education about research, if people do not understand what research is about, they will not take part. Education first—taking part later. (South Asian Men)
	Lack of understanding of what prevents people from accessing healthcare	Many patients go to see the GP as a last resort, not the first port of call. Need to understand why this is. Are there access barriers? It's difficult to speak on behalf of other people. Health services access and urgent care isn't always the best. (Retired GP)
	Research outcomes not shared with communities	…we want quick answers, what is the outcome, the results now? We are not interested in long‐term things. Because of this reason, some people do not want to be involved. (South Asian Men) Researchers in community centres, places of worship, but reports are never shared and researchers do not come back… Outcomes are never disclosed to the community. (South Asian Men) People from ethnic minority groups want ethical treatment. They don't want to be patronised, and they want to know that they're contributing. (Midwife)
	Past experiences and perceptions	Recently, I heard about men being involved in research and they died (young men died). Money is not good for you if you are dead. (African Caribbean)
Motivation	Helping others	I will take part in research if this helps people, even medical/hospital‐based research. During Covid‐19, if they will say to me—take 10 jabs, I will take it. (African Caribbean) In a personal experience, vouchers and incentives don't play as the number one motivator that people care about. They care about their contribution and helping others as long as helping others is clear. (Researcher Paramedic)
	Topics of importance to individuals and communities	If it relates to me or have impact on my personal situation—pull in factor. It matters to me. (Eastern European) Topic is important—personal experience can help. Relevance/when it hits home—it matters to me. (GT)
Increase research participation	Empower communities	Training people from the community to help with the research… Empowering the community to train community members. Once you train people, people can do it in the community. (Somali) We need to build long term with people in communities and engage with people on their terms. (Retired GP)
	Consider community circumstances and how health information is sought	…understanding people's circumstances is also an important thing. Yes, I agree most of our community, ethnic minorities they do not want to take part in research because if people have bad health condition they can find the solution from their GP or somewhere else, so they do not think it will affect them. (South Asian Men)
	Provide incentives for taking part	… for some people, to engage in research, an incentive will be important like vouchers, other incentives. (South Asian Men)
	Share research details and findings with communities	I have previously taken part in PhD research. I felt valued and my opinions mattered. It was nice to see the outcomes of the research involvement… (Retired GP)
	Uphold respect and value for community participation	… commended for the PPI and so is it was inspiring to see that work being acknowledged. And so now putting a PPI plan in place is more than just tokenistic. Why don't you do it as it makes a big difference to the research? (Carer)
	Use alternative approaches to engage with people	Using door‐to‐door approach, especially for people who do not leave their home…GP promotion‐ GP could offer to their patients if they want to take part in research. (Somali) People exposed or affected by certain conditions may be more likely to partake in research work. It's about finding a way to approach people. (Nurse) Practical arrangements/location of research/who is in the room. Depends on who is in the room from the community, people may not want to speak up/share things in front of them/open up. (GT) …the latest invitation I received inviting me to take part in cancer research‐related study, it asks for a blood sample. It states that this can be done in various locations (including mobile points, GP). This is convenient for me (Eastern European) I prefer to be involved in face‐to‐face activities; you can raise your hands; zoom can be tricky. (African Caribbean) A short questions/survey/links online are good options to encourage more people… A demo/short clip‐ who different types of research look like? What to expect. (Eastern European)

Abbreviations: GP, general practitioner; GT, Gypsy Travellers.

*Understanding of health research*: varied widely across all the communities, and there was overall low research participation history. Some participants had good perceptions of the purpose of research, including positive concepts from past experiences, such as research focused on investigating problems related to health and communities towards finding solutions and new information for specific groups.

*Negative research perceptions*: were reported by those often afraid of getting involved. For instance, more African Caribbean participants relayed negative opinions of research, including the perception that they were being used as ‘guinea pigs’ and being used with no benefit from participating in research. These experiences made some groups more reluctant to participate in research.

*Barriers to participation*: was commonly related to lack of education, poor understanding of different groups' participation needs, past experiences, and not having access to research findings after research completion. For instance, participants shared expectations they wanted to see when participating in research, including being respected and provided with research outcomes and impact related to their community.

*Motivation for research participation*: was primarily to support the search for identifying health solutions that could benefit the communities. Although incentives were considered an important motivating factor, it was stated not to be sufficient encouragement to participate in research.

*Increase research participation*: was best thought through community involvement and empowerment. This includes considering unique community circumstances and cultural values, how health information is sought and alternative communication challenges, for example, engaging community religious leaders. Increasing research participation is also associated with increasing the confidence of community members in the care services.

#### 3.3.2 Research recommendations

Based on the ethnic minority groups in the study, the most common health research areas of interest for the representative groups were cancer, diabetes, hypertension/high blood pressure, mental health, sickle cell and health promotion. While the common social care research priorities included early care interventions, social isolation, substance misuse and understanding the needs of communities. For care providers, training, improving communication and awareness of different communities, linguistic support, and referral services were identified as core areas to explore. A detailed summary of the recommended research areas by the different groups represented in the study is presented in File S2.

## 4 DISCUSSION

This qualitative study explored the health and social care experiences and future research priorities of ethnic and other minority populations in the East Midlands. Challenges to care service access were mainly linked to access delays, discrimination, poor staff competency skills, and information and language barriers. The findings showed that individual literacy, language and urgency influenced information‐seeking actions. The most influential information sources were local community organisations and word‐of‐mouth. The primary health concerns included chronic and mental health issues. Early interventions were emphasised as crucial, but miscommunication and language barriers often created hesitancy in seeking care. Cultural restrictions also hindered engagement in certain health activities and difficulty accessing accurate information and support. Fear, distrust, misconceptions and geographic location further influenced this.

Perceptions of research were mixed, but some had good knowledge of different types of research. Research barriers included trust, privacy, language, fear of stigmatisation, lack of understanding, not receiving feedback from previous research involvement and negative past experiences. Recommendations to increase research participation included understanding primary motivations for participation, empowering communities to lead research activities, considering communities' circumstances, providing incentives, sharing research details and findings with communities, respecting the value of community participation and utilising different engagement approaches. Suggestions to increase awareness of and access to health services for ethnic minorities included improving communication, access, continuity of care, community services, cultural competence, community representation, trust and health system structures.

Several studies have identified similar challenges faced by ethnic minority populations accessing health and social care services.^9^, ^22^, ^23^ These include structural and cultural discrimination, racism and social determinants of health such as socioeconomic status, housing, occupations, a higher burden of diseases and cultural barriers.^22^ Evidence has also shown that discrimination is a fundamental cause and driver of adverse health outcomes in ethnic minorities and health inequalities.^9^, ^24^ Such challenges contribute to worse health outcomes, lower life expectancy and wider disparities, even among children.^24^, ^25^, ^26^, ^27^ A systematic review found multiple associations between self‐reported discrimination and health, and this included associations with poorer mental health, higher chronic disease incidence and preclinical indicators of disease, poor health behaviours and lower use of healthcare services and adherence to medical regimens.^28^ Ethnicity impact on health outcomes in the United Kingdom is shown among Black women, who are nearly four times more likely to die during pregnancy than White women, and most of them had severe and multiple disadvantages, including mental health concerns.^26^

Cultural racism, discrimination and marginalisation have been shown to be motivated by stereotyping, which can give rise to unconscious bias and stigmatisation.^24^, ^29^, ^30^, ^31^ Mental health‐related stigma, mostly among men, was of particular concern. Cultural stigmatisation was reported as the main barrier, and this was generally associated with poor awareness, similar to findings in other studies.^32^, ^33^ Bias from health providers has also highlighted stereotyping and cultural insensitivity, which contributed to negative experiences, created barriers, hindered access to healthcare and influenced healthcare‐seeking behaviours of ethnic minorities.^30^ Reported stereotyping of mental health among Black people supports the evidence of the greater risk of detention of Black people under the Mental Health Act than White people.^34^ This experience further discourages people from presenting mental health challenges to health and social care providers.

Other studies have also observed a high appreciation for pharmacy services among ethnic minorities.^35^, ^36^ A UK study showed that ethnic minority members appreciated the supportive role of community pharmacists and how these break down barriers to health service access, increase health awareness and build trusting relationships.^37^ Community pharmacists have also been reported as the most accessible primary care HCPs, and the advantages they provide include medicines consultations, quick access without booking appointments, and convenient timing.^35^, ^37^, ^38^ Another health service preference identified was receiving care from health staff from ethnic minority communities as well as obtaining information through community organisations. Other studies have also observed similar preferences among other ethnic minority groups. An advantage of having ethnic minority staff, in addition to improving access to care, is the potential to overcome language and interpretation barriers, which is a significant challenge among ethnic minorities.^35^

The common health and social issues reported reflect the general health burden of ethnic minorities in England,^39^ and these were also mirrored in the suggested research priorities. Identifying effective interventions to tackle the challenges requires tailored, culturally relevant, and acceptable strategies. This can be achieved through research to test, compare and determine the best strategies for preventing and treating disease and informing health policy. A one‐size‐fits‐all approach is inadequate as barriers identified vary between community groups. To ensure health policies serve diverse ethnic minority populations, all ethnic and cultural groups must participate in health and social care research. For instance, addressing the child development concerns raised will be understanding the unique experiences and cultural perspectives of different ethnic minority groups.^27^, ^40^ Advantages of high‐quality early childhood programmes go beyond direct health benefits and have a social impact, such as reduced crime, higher economic earnings and promoted education.^40^ Furthermore, tailored community initiatives can build community cultural and racism issues and have potential health benefits.

Similar to the findings in this study, other studies have highlighted the willingness of ethnic minority groups to participate in research if the study has direct relevance to them and their community, if they are approached with respect and sensitivity, and if they are presented with clear explanations of the research.^6^, ^41^ However, evidence has also shown that the involvement of ethnic minority groups through different stages of the research trials is inconsistent.^2^ A review of clinical trials shows that most studies do not report data on research inclusion approaches, screening, follow‐up or outcome effect estimates of a specific ethnicity.^12^, ^42^, ^43^ Factors that have limited ethnic minority representation in research have included stereotypical and negative attitudes of researchers, language skills, lack of knowledge of the study and research processes, relevance of research and study protocol, time, resource constraints (e.g., travel costs), lack of diversity within the research team and inadequate housing or transport.^43^ Most of these barriers are at an individual level,^8^ but other barriers identified at the healthcare system and hospital level include restrictive study designs, financial costs associated with running trials, doctor–patient relationships and lack of community engagement.^43^, ^44^ Overcoming these barriers is key to improving recruitment and participation in health and social care research, and tailored strategies will need to be implemented to improve participation in research of ethnic minority groups. A one‐size‐fits‐all approach will be inadequate as barriers can vary between different communities.^8^ Considering the recommended research areas of the different ethnic minority populations would encourage their interest and increase participation. Thus, minority‐focused research study development and implementation need to be explored further. Also, a comparative analysis of the participant groups in this study could be further explored at a national and international level to understand better how the views differ across different cultures and geographically.

### 4.1 Strength and limitation

This study presents a qualitative account of ethnic minority populations' health and social care experience, priority areas for future research and preferences for research involvement. Strengths of the study include the diversity of participants, the co‐production of the study with ethnic minority communities and care providers, and the novel insights gained from different participant perspectives (ethnic and social minorities), providing a rounded account of ethnic minority care experiences. This study also contributes to previous research findings that prioritise minority groups, their research interest and good practice for improving participation in research.

The authors recognise that the participant sample was limited in representation from selected ethnic minority communities residing in the East Midlands region in the UK; hence, the findings may not be generalisable to other regions and countries. Future research will be needed to explore other ethnic groups and regions to broaden our understanding of the health and social care needs of more minority groups. Nevertheless, the findings were reflective of other ethnic minorities in the UK. Follow‐up research could also revisit some of the emerging themes from this research to determine areas of research engagement with different community groups.

## 5 CONCLUSION

This co‐produced study describes the health and social care experiences and needs of different ethnic minority groups and their research experience, interests and expectations. Poor access to services and research under‐representation are complex and could be primarily attributable to cultural discrimination, stigmatisation and hesitancy on the part of communities, lack of adequate support and other socioeconomic factors. The research opportunities and recommendations shared in this study can be used to improve access to care for people from ethnic minority communities. The broad‐reaching recommendations generated in this study could be adopted to overcome barriers for people from ethnic minority communities worldwide and inform the design and implementation of interventions and research.

## AUTHOR CONTRIBUTIONS

**Winifred Ekezie**: Conceptualisation; investigation; writing—original draft; methodology; validation; writing—review and editing; formal analysis; project administration; data curation; resources; supervision; funding acquisition; visualisation; software. **Shabana Cassambai**: Conceptualisation; investigation; funding acquisition; writing—review and editing; validation; data curation; resources; project administration; writing—original draft; visualisation; formal analysis; methodology. **Barbara Czyznikowska**: Conceptualisation; investigation; funding acquisition; project administration; resources; writing—review and editing; validation. **Ffion Curtis**: Conceptualisation; investigation; funding acquisition; writing—review and editing; validation; methodology; project administration; supervision; data curation; formal analysis; software; resources. **Lauren L. O'Mahoney**: Conceptualisation; investigation; funding acquisition; writing—review and editing; resources; project administration; validation. **Andrew Willis**: Conceptualisation; funding acquisition; writing—review and editing; resources. **Yogini Chudasama**: Funding acquisition; writing—review and editing; resources. **Kamlesh Khunti**: Conceptualisation; funding acquisition; validation; supervision; writing—review and editing. **Azhar Farooqi**: Conceptualisation; funding acquisition; writing—review and editing; project administration; supervision; resources; investigation.

## CONFLICT OF INTEREST STATEMENT

Kamlesh Khunti is the Chair of the Ethnicity Subgroup of the UK Government Scientific Advisory Group for Emergencies (SAGE) and a member of SAGE. The remaining authors declare no conflict of interest.

## Supporting information

Supporting information.

Supporting information.

## Data Availability

The data that support the findings of this study are available from the corresponding author upon reasonable request.
