# Supplementary material for: Health and social care experience and research perception of different ethnic minority populations in the East Midlands, United Kingdom (REPRESENT study)
Source: Health Expect. 2023 Dec 21;27(1):e13944. doi: 10.1111/hex.13944 (PMC10733974; doi:10.1111/hex.13944)
Supplement: Supplementary file 1 — Supporting information. [file HEX-27-e13944-s002.docx]

# Health and social care experience and research perception of different ethnic minority populations in the East Midlands, United Kingdom (REPRESENT Study).

**Supplementary 1**: Topic guide for Health and social care research priority setting among ethnic minority populations

**Health care experience**

- How do you generally receive health information?

Here we mean general health messages such as those we all heard during the pandemic around restrictions and vaccinations, and others like information about healthy eating or giving up smoking.

*(****Prompts****: Did you see adverts on the TV, social media or word of mouth?)*

- - What has been your experience getting information in that way? And are there any better ways you can think of?
  - What do you think is the most effective way for you, your family and your community to receive such health information?
- Thinking about times when you or your family had to seek medical attention

*(****Prompt:*** *For example, GP, hospital clinics, pharmacists, dentists)*, could these experiences have been better? Is there anything that stands out in your mind?

This could be a good or bad experience; perhaps you felt rushed or that the healthcare professional did not listen to you or understand your concerns adequately.

- - If anything, what could have improved this experience?
  - What would you do differently now (if anything)?
  - What would you have wanted that you didn't feel you received?

**Health and social care concerns**

- What are the most important health and social care concerns **for you and your family**? *(****Prompts:*** *For example, specific health conditions, health services, social care and support – define social care context)*
- Do any other health and social care topics worry you, especially thinking about the diversity in **ethnic minority communities**?

*(****Prompts:*** *For example, specific health conditions, health services, social care and support, support for asylum seekers who fear deportation, understanding community /individual confidence in accessing healthcare)*

- - Do you think there are important health care needs that are currently overlooked in the UK?
  - What are the most important areas you believe could improve the health of ethnic minority communities?
  - What specific improvements would you like to see for those issues?

**Health research**

- What is your understanding of health research? (please could you expand on your answer and examples).
- Do you see many opportunities to get involved in health research, and if so, where do you see these opportunities?
- Have you ever taken part in health research?
  - If yes, was it a positive or negative experience?
  - If you are comfortable, please elaborate.
  - If no, have you ever been asked?
  - Would you participate if asked?
- In general, do you think research is important to improving health?
- What concerns do you have about participating in research?
- What are some of the barriers to participating in research?
- What health concerns would you like to see being researched or addressed?
- What would you suggest are important areas to explore in a research study about the health and wellbeing of ethnic minority communities?
- If you had to rank health topics that are important to you, what would be the top 3 significant research priorities (you feel needs to be addressed for different ethnic minority communities, remembering that these will be used to inform future health and social services? This could be a health topic that matters to you and/or your community or one that you feel your current healthcare service doesn’t cover enough already?

**Recommendations**

- What health conditions do you think would be most useful to explore in our research?
- Do you have any suggestions about how we could get more of our community members involved in research?
- What do you think the main barriers are stopping people from taking part in research – these could be from not knowing about projects to things related to how we speak about research and how we recruit and conduct the different stages?
- What are the things we are doing well to involve our communities in research (if any!)?
- What would ethnic minority communities expect from their involvement in research? *(****Prompts****: incentive, recognition, discount services, access to study resources and technology, 1-to-1 support, regular follow-up, updates and reports)*

**Non-community members’ specific questions:**

- What are the most under-researched and should be priority areas for research on ethnic minority communities?
- What is needed to be promoted to raise public awareness about research among ethnic minority communities?
- What relevant outcome indicators should be measured to demonstrate the success of any health and social care research and intervention among ethnic minority communities?

**Researchers’ specific questions**

- What are your suggestions on how to improve engaging ethnic minorities in research?
- Can you share some successful community research engagements you have conducted?

**Healthcare providers’ specific questions**

- What communities do you feel are underserved by services?
- What types of services are under-utilized by ethnic minority communities? And what suggestions would you make to improve their uptake?
