# Supplementary material for: Health and social care experience and research perception of different ethnic minority populations in the East Midlands, United Kingdom (REPRESENT study)
Source: Health Expect. 2023 Dec 21;27(1):e13944. doi: 10.1111/hex.13944 (PMC10733974; doi:10.1111/hex.13944)
Supplement: Supplementary file 2 — Supporting information. [file HEX-27-e13944-s001.docx]

# Health and social care experience and research perception of different ethnic minority populations in the East Midlands, United Kingdom (REPRESENT Study).

**Supplementary 2: Summary of the recommended research areas by the different groups**

| **Groups** | **Community** | **Top research areas recommended** |
| --- | --- | --- |
| **Ethnicity** | **African Caribbean** | - Blood pressure - Communications/awareness amongst HCPs on sickle cell condition affects - Diabetes - Mental health - Prostate issues - Sickle cell |
|  | **Eastern European** | - Cancer (breast and womb in women; prostate and lung cancer in men) - Child development and health (genetic conditions) - Communication with HCPs (time spent with patients/ holistic approach) - Dental access and care - Elderly care/support - Emotional and mental health care/support - Primary care (better patient/doctor relationship; access issues) - Psychological support (support/ access therapy) - Psychological support for adults (including the older generation) and children, feeling of loneliness - Referrals services - Social isolation - Social isolation and social care. - Substance misuse - Understanding people’s needs- mapping up and acting on the key issues - Women’s health (pregnancy/post-natal period/cancer) |
|  | **Gypsy Travellers (GT)** | - Cervical cancer - Diabetes - Healthy lifestyle awareness - Mental health and men |
|  | **Somali** | - Arthritis (old age and frailty) - Blood pressure - Cancer (screening, tests, awareness, management, - Diabetes - GP delays - Health and wellbeing education - Kidney problems - Mental health (children and young people) - Pharmacy health checks - Pharmacy service communication to the community - Referral to specialist - Vitamin D deficiency - Women's health (childbirth) |
|  | **South Asian Men** | - Awareness about health conditions and available support - Cholesterol - Community consultations (identify people’s needs) - Community facilitators to provide linguistic support, help people, share information – work with NHS and people’s needs) - Diabetes - Education on chronic long-term conditions, organ donation - High blood pressure - Language support - Raise awareness about taking part in research - Workforce representation |
|  | **South Asian Mixed** | - 111 awareness on how to speak to ethnic minority communities - Building trust in ethnic minority communities within healthcare - Dementia (for patients and carers) - Mental health - Timely remuneration - Engage ethnic minorities, children and grandchildren in dissemination of health information |
|  | **South Asian Women** | - Access to exercise and information about maintaining good physical and mental health. - Addictions (mental health, family breakdowns) - Cancer - Dementia - Diabetes - GP-hospital-dentist (access) - Health and social care for the elderly - Heart problems - Mental health (support, prevention, management) - Promoting good health - Remove barriers to accessing social care. |
| **Other types of minorities** | **LGBTQIA+** | - Carers support (mental health and wellbeing; strategic planning; lack of support; not being seen as a patient; carers inclusion across health and social care support) - Finding the right services/improving general knowledge about service availability - Health education and targeted education on sickle cell, drugs and alcohol - Healthy lifestyle/eating on the budget - Improving communication/information sharing between health and social care services - Personality disorder (treatment/medication) - Recognising the impact of caring burden on emotional wellbeing/needs |
|  | **Refugees & Asylum Seekers** | - Access to GP - Accessibility (Translators, Interpreters) - Communication (keep people informed) - Diet and new arrivals/nutrition - Helpline to call to check what to do - Mental health - More attention to patient’s needs - Patient reassurance in A&E - Social networks (having people/friends, peer support, supporting new arrivals, building networks) - Understanding support and access - Waiting times at hospitals - Women’s’ health (rape/ trauma/abuse) |
| **Healthcare providers** | **Carer** | - Cardiac - Education (e.g., using A&E visits, triage) - Effect between cardiac and mental health - Mental health - Patient influence – medically and outside (doctors learning both sides) |
|  | **GP** | - Diabetes. - Education - Health promotion (exercise and better diet awareness) - Hypertension - Kidney disease - Sickle cell disease - Support and facilities - Vitamin D deficiency |
|  | **Midwife** | - Cancer - Communities to disseminate information instead of HCW using community settings - COVID - Diabetes - Pandemic vaccination (vaccines reluctance) - Sickle cell services - Social experiences |
|  | **Nurses** | - Breast cancer - Cancers - COPD - CVD - Diabetes - Diet - Heart failure - Hepatitis C - Hypertension, especially familial hypertension - Interstitial lung disease (communication) - Lack of certain healthcare services, such as for cancers, heart failure, hypertension, and COPD, particularly in the Polish community. - Liver cirrhosis (alcohol and non-alcoholic driven) - Progressive super neurologic palsy - Respiratory illnesses |
|  | **Researcher Paramedic** | - Closed-off communities not accessing emergency care (e.g., Chinese community) - Dental care - Mental health (penetrate closed communities, e.g., GT communities) |
|  | **Retired GP** | - Access barriers - Alzheimer - Dementia - Early interventions - Elderly healthcare (lack of understanding from HCPs) - HCP knowledge sharing and service delivery re-evaluate - Health services access and urgent care - Improve HCP understanding of different communities. - Mental health service |
